# Psychological and physiological effect in humans of touching plant foliage - using the semantic differential method and cerebral activity as indicators

**DOI:** 10.1186/1880-6805-32-7

**Published:** 2013-04-15

**Authors:** Kazuko Koga, Yutaka Iwasaki

**Affiliations:** 1 Graduate School of Horticulture, Chiba University, 648 Matsudo Matsudo-shi, Chiba, 271-8510, Japan

**Keywords:** Cerebral blood flow, Complementary and alternative medicine, Nature, NIRS, Plant foliage, Pothos, Semantic differential method, Sense of touch, Tactile, Therapy

## Abstract

**Background:**

Numerous studies have reported on the healing powers of plants and nature, but there have not been so many instances of experimental research. In particular, there are very few psychological and physiological studies using tactile stimuli. This study examines the psychological and physiological effects of touching plant foliage by using an evaluation profile of the subjects’ impressions and investigating cerebral blood flow.

**Methods:**

The subjects were 14 young Japanese men aged from 21 to 27 years (mean ± standard deviation: 23.6 ± 2.4). With their eyes closed, the subjects touched four different tactile samples including a leaf of natural pothos (*Epipremnum aureum*). The physiological indices were compared before and after each stimulus. Psychological indices were obtained using a ‘semantic differential’ method.

**Results:**

The fabric stimulus gave people ‘soft’ and ‘rough’ impressions, ‘kind’, ‘peaceful’ and ‘pleasant’ feelings psychologically, and a sense of physiological calm. On the other hand, the metal stimulus gave people ‘cold’, ‘smooth’ and ‘hard’ impressions and an image of something ‘artificial’. The metal stimulus caused a stress response in human cerebral blood flow although its evaluation in terms of ‘pleasant or unpleasant’ was neutral. There were no remarkable differences between the stimuli of natural and artificial pothos compared with other types of stimulus psychologically. However, only the natural pothos stimulus showed a sense of physiological calm in the same appearance as the fabric stimulus.

**Conclusions:**

This study shows that people experience an unconscious calming reaction to touching a plant. It is to be concluded that plants are an indispensable element of the human environment.

## Background

The artificial environments in which modern human beings live have produced many kinds of stress. In particular, unfavorable economic conditions in recent years have resulted in increased mental stress. As a result, the world has been flooded with various therapies. These therapies are not only used for relaxation or release from tension in daily life, but also as complementary and alternative medicine (CAM) to the medical treatment of and rehabilitation from illness and physical and mental handicaps [1]. There are several reasons why CAM has been the focus of increasing attention in modern society, including the limitations of Western medicine in treating chronic diseases and stress-related disorders, rising medical expenses caused by low birth rate and longevity, and increased levels of patient interest in the selection of medical treatment [1]. However, there is little significant evidence for CAM. Significantly more medical and scientific evidence is required in order for CAM to be better utilized to improve quality of life and activities of daily living.

Many types of CAM bear some relation to plants. According to Imanishi [1], a search of MEDLINE with the keyword ‘CAM’ in 2007, indicated that 41% of CAM was ‘phytotherapy’, while 15% of CAM could be termed ‘medicine-traditional’, a form of medicine that uses pharmaceuticals and oils made from plants, such as kampo (Chinese traditional) medicine and Ayurveda. This suggests that plants are indispensable in healing.

Recently there has been an accumulation of medical and scientific evidence in this discipline [2]. However, the healing mechanisms of plants or natural environments are not clear, because people and plants are both complex systems. The different dimensional effectiveness of plants and nature has not been clarified and this remains a barrier to clearly understanding their healing mechanisms. In any case, as a minimum indicator of their healing mechanisms, it is important to show the psychological and physiological effects of plants and nature. Many studies on the therapeutic power of plants and natural environments have been conducted [3-5]. These studies show the restorative effect and reduction in stress levels that come about through nature, regardless of their varied theoretical backgrounds [6]. One recent study reports that positive affect increased and anger decreased after 50 minutes of walking in a nature reserve; the opposite pattern emerged in an urban environment [3]. Forest environments in particular have been the focus of ‘forest therapy’ or ‘forest bathing’ in Japan. Many studies of forest therapy have been conducted. These studies report that various elements of forest environments cause people to relax physiologically and psychologically [7-9]. Recently, the influence of nature therapy has been the subject of much attention from an epidemiological viewpoint and studies show a link between green spaces and the rate of disease [10].

Horticultural therapy is one of the most popular therapies utilizing the healing power of plants. Horticultural therapy was originally developed for the mental health care of returned soldiers in the United States. Nowadays, horticultural therapy has been used with various target groups, including psychiatric patients [11], dementia patients [12], older people [13], children [14], and prisoners [15]. The many reports conducted show the mental, physical, and social effects of horticulture therapy. However, most of these reports come from anecdotal evidence collected in various settings. A clear causal relationship between people and plants has not been discussed, since many complicated factors are involved [16].

More experimental research is necessary to clarify the healing mechanism of plants. How do people detect stimuli using all five senses? How do plants influence peoples’ minds and bodies? The majority of studies on the effect of stimuli concentrate on the visual sense [17-19]. Ulrich [18] examined the recovery rates of patients who underwent gall bladder surgery and found that those patients who had a natural scene to view recovered faster than those patients who viewed an urban scene. Kaplan showed that a view of nature from the window contributed greatly to the residents’ wellbeing [17]. Moreover, there has been a gradual accumulation of experimental studies of the way in which the visual stimulus of nature and plants is reflected in the human mind and body [20,21].

There has also been a gradual increase in the number of studies of the effects of odor, such as the use of aromatherapy, and these studies shed light on the pharmacological and physiological effects of essential oils used experientially [22,23]. There have been more sophisticated studies of the effects of lavender, which is very well known as a traditional holistic relaxant. These studies reported an increase in parasympathetic modulation in middle-aged women with insomnia [24], the relaxing effect of lavender on patients undergoing cosmetic procedures [25], and a significant improvement of agitated behavior in severe dementia patients [26].

However, there are not so many studies regarding the other senses. The tactile sense has been especially neglected, in spite of its importance in human emotion.

Although the skin is the only sense organ that recognizes the real world [27], few studies deal with the tactile (the sense of touch). The skin’s development originates in the same way as the brain and that is why it is sometimes referred to as ‘the third brain’ [28]. The skin is an organ that possesses higher functions, such as cognition and judgment [28].

Moreover, it is said that there is a close relationship between tactile stimuli and brain chemistry, such as oxytocin and serotonin [28,29], which manages the sociality of human beings. The latest research also shows strong relationship between touch and emotions [30].

Above all, the skin separates and distinguishes between human beings and the environment. The mind was not born without existence of the skin [31].

Previous studies on the tactile sense and plants are limited. Abe and Masuyama [32] focused on the tactile qualities of wood as a material. Yamada [33] investigated the qualities of residence, and wood has excellent qualities for fit habitation. Miyazaki *et al*. [34] and Miyazaki and Morikawa [35] reported that touching wood gives one a relaxed feeling while touching metal induces a stress reaction. In this way, some previous studies dealt with the tactile nature of wood as a material, but only a few studies focused on empirical evidence that touching living plants gives people a feeling of comfort and relaxation [36,37].

Animal-assisted therapy is known as a therapy that utilizes living creatures. Plant-assisted therapies, such as horticultural therapy, are very similar to animal-assisted therapies in that a social effect is expected through prolonged activity. On the other hand, the effect of short-term tactile stimuli of animal-assisted therapy is acknowledged [38,39], but the effect of short-term touching of plants is not.

Therefore, the aim of this study is to clarify the psychological and physiological effect of touching living plants in the laboratory. Since many previous studies show that plants and nature can have both recuperative and relaxing effects, it is to be expected that touching plants can also have psychological and physiological effects. Although the physiological mechanism of such theories advocated by Ulrich and Kaplan and Kaplan is not fully clear, it seems that there may be a positive influence on the immunological system as a result of lessening strain in the sympathetic nerve. In some past studies, Miyazaki has mentioned that activity in the prefrontal area was calmed by the olfactory stimulus of wood and the forest environment [40]. Therefore, the same change would be anticipated in this study through the stimulus of touching plant foliage. This understanding will help the fundamental understanding of the relationship between people and plants and contribute to the optimum uses of plants and green spaces in urban areas.

## Methods

### Subjects and stimuli

The subjects were 14 young Japanese men aged from 21 to 27 (mean ± standard deviation: 23.6 ± 2.4). The subjects gave informed consent to the experiment in accordance with the protocol reviewed and approved by the Human Investigation Committee of Chiba University.

**Figure 1:**
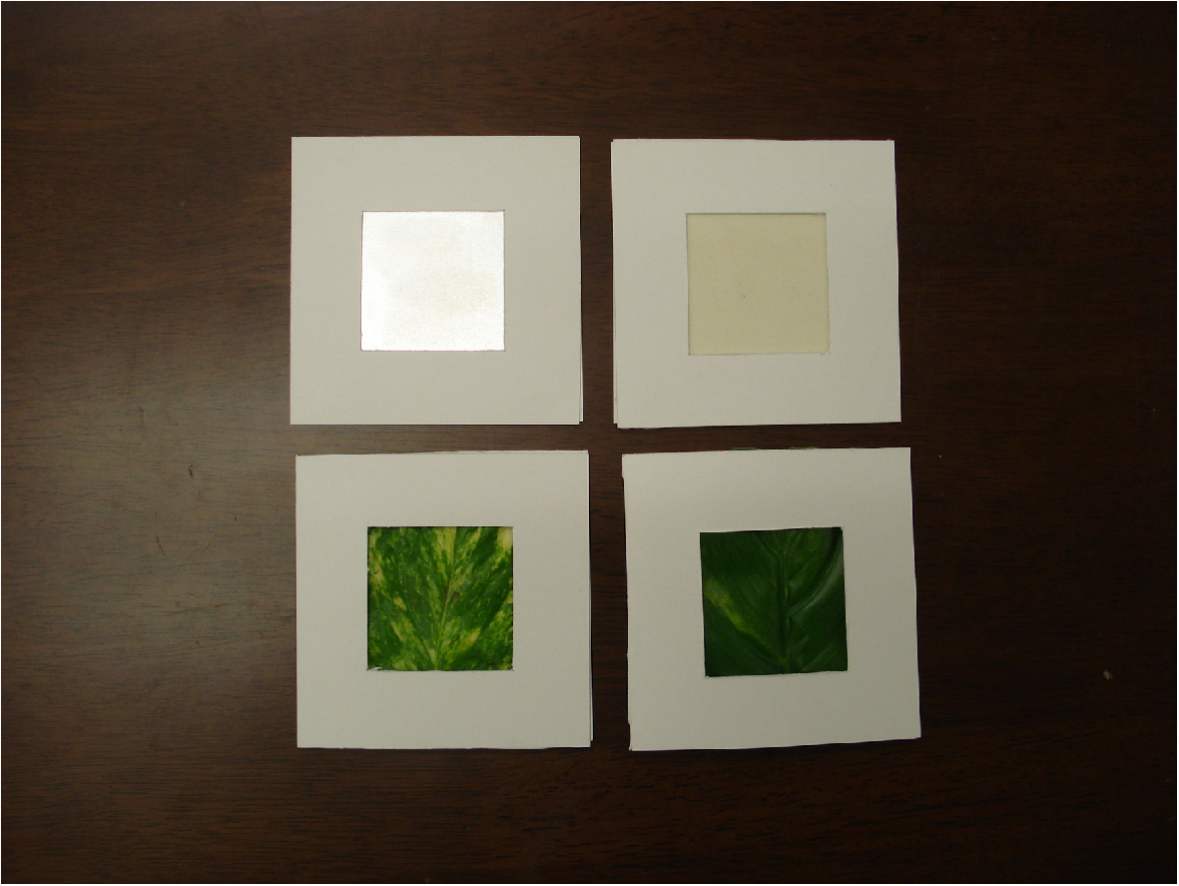
**Tactile samples.** The samples are, clockwise from top left, metal, fabric, artificial pothos, natural pothos.

Four different tactile experimental samples were prepared: a plate of aluminum, a piece of velveteen, a leaf of natural pothos (*Epipremnum aureum*), and a leaf of artificial pothos made of resin. Each material was held between two pieces of 10cm × 10cm square paper that had a 5cm square window cut out in front to make a planar sample (Figure 1). The sample of natural pothos was checked for quality after every session, and changed for a fresh leaf if it had been scratched or had noticeably dried.

### Data collection

**Figure 2:**
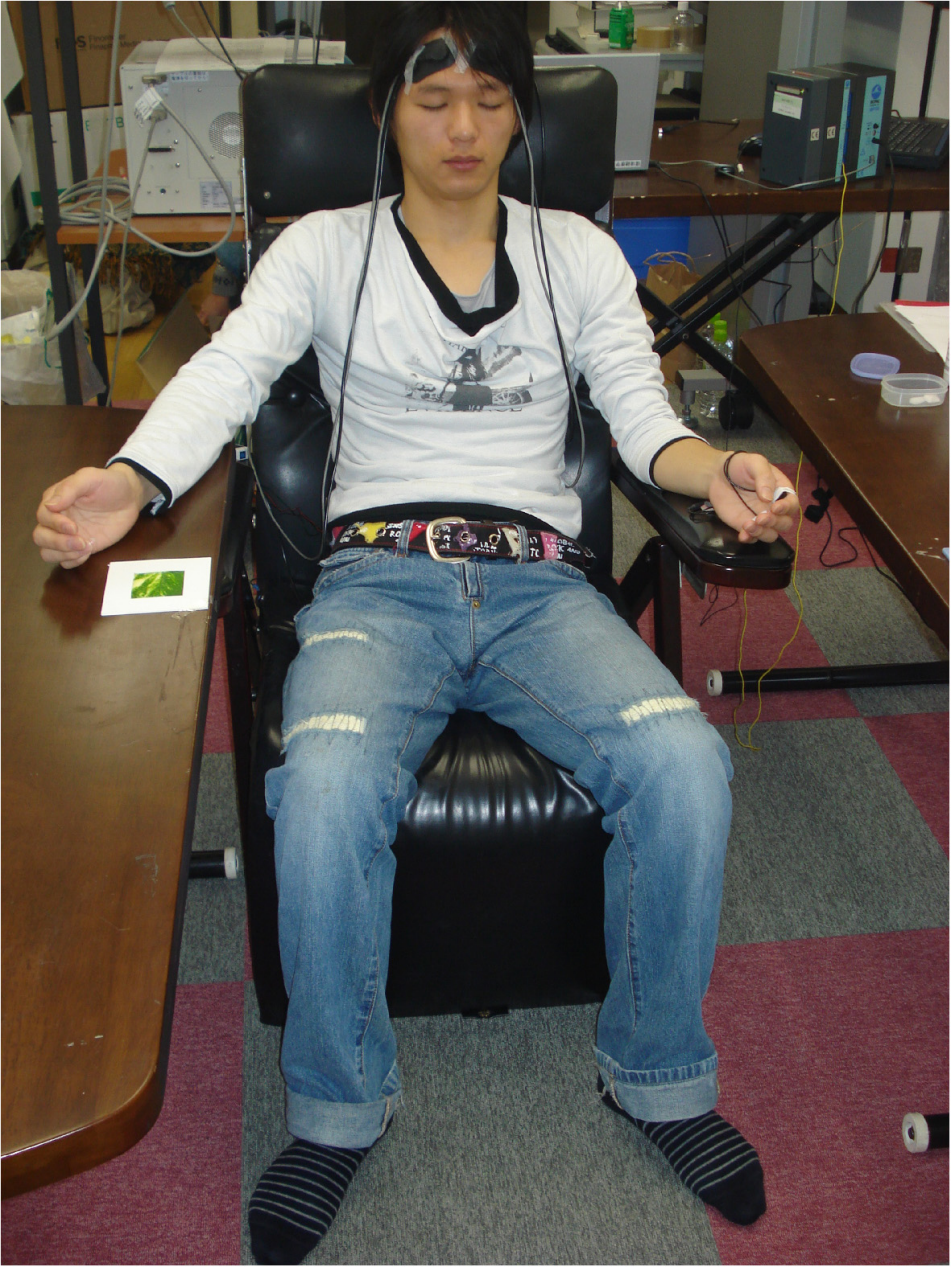
Subjects.

Subjects were seated on a chair while closing their eyes and putting their right hand on a table in front of them (Figure 2). The subjects rested their wrist only on the table with their fingers held lightly in the air above the table. They were then instructed to touch the sample with their fingers. The measuring devices were placed behind the subjects.

Physiological data were acquired, showing a concentrated change in cerebral blood flow (CBF) as an index of central nervous system activity. Cerebral function can be evaluated by measuring the activity of a nerve cell directly or by measuring the change in CBF accompanying nerve activity [41]. In recent years, optical topography using near infrared spectroscopy (NIRS) has attracted much attention from the point of view of time resolution, spatial resolution, and above all, from the point of view of the ease of measurement. Although NIRS was initially developed for diagnosing brain ischemia and low oxygen, it turns out that a change in CBF and oxygen metabolism accompanying nerve activity can be measured as a secondary signal, and that these signals can be applied to various fields as a method of cerebral function imaging [42]. Although there is some disagreement about the extent of correspondence between nerve activity and substance movement, a similar nerve phenomenon is shown in previous research, which carried out simultaneous recording of electric activity and fMRI in the visual cortex of an ape [43]. The CBF was surveyed using near infrared spectroscopy by attaching two channel sensors on the right and left sides of the forehead (NIRS; NIRO-300; Hamamatsu Photonics, JAPAN). These locations in the brain play a critical role in higher cognitive functions, concentration, and emotion [44,45].

Psychological data were acquired using a semantic differential (SD) method to evaluate the profile of the subjects’ impressions, and ten pairs of adjectives were used for SD (Table 1).

**Table 1:** Pairs of adjectives used in semantic differential method

**Pairs of adjectives in English**	**Pairs of adjectives in Japanese**
warm, cold	あたたかい-つめたい
smooth, rough	つるつる-ざらざら
soft, hard	やわらかい-かたい
natural, artificial	自然な-人工的な
familiar, unfamiliar	親しみやすい-親しみにくい
kind, unkind	やさしい-やさしくない
calming, stimulating	沈静的な-覚醒的な
peaceful, anxious	安心な-不安な
pleasant, unpleasant	快適な-不快な
like, dislike	好きな-嫌 いな

**Figure 3:**
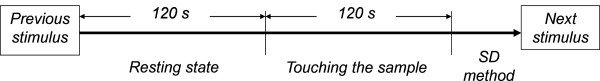
Experimental protocol.

After the subject rested on a chair, CBF was recorded as ‘pre-stimulus’ for 30 seconds. Then according to the instructions, the subject touched the sample, after which the CBF was recorded as ‘post-stimulus’ for 120 seconds. The semantic differential was investigated after touching the sample. The subjects closed their eyes while conducting this experiment, but not while answering the SD questions. In this experiment, metal was used as the control and the samples were presented at random. Figure 3 shows the experimental protocol for this study. The experiment was carried out in the shielded room at Chiba University from 15 to 20 December 2010. The average temperature, the average humidity, and the average illumination factor were 21.2 ± 1.8°C (mean ± standard deviation), 54.7 ± 4.7% and 1050.0 ± 89.7 lux, respectively.

### Data analysis

The CBF was recorded every one second and analyzed as oxygenated hemoglobin. Based on the contact time (*t*_0_*=* 0 seconds), data from −30 seconds to 0 seconds were averaged and used as the pre-stimulus. The difference between each post-stimulus and its corresponding pre-stimulus was analyzed by Student’s *t* test paired comparison. Steel-Dwass’s multiple comparison test was applied after one-way analysis of variance (ANOVA) for the comparison of SD scoring.

## Results

### Impression profiling evaluation

**Figure 4:**
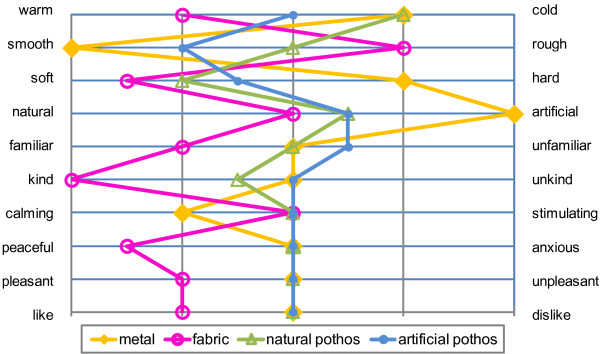
**Result of the evaluation profile of the subjects’ impressions using the SD method.** These plots show median SD score.

In the results of the SD method, the fabric was significantly warmer, rougher, softer, and more peaceful than the metal, the natural pothos, and the artificial pothos. The metal was significantly colder than the artificial pothos (*P* < 0.01; Figure 4, Table 2). Also, the fabric was significantly warmer than the metal (*P* < 0.01), the natural pothos (*P* < 0.05), and the artificial pothos (*P* < 0.01). The metal was significantly smoother and harder than the natural and the artificial pothos. In the ‘natural or artificial’ item, the metal was significantly more artificial than the fabric, the natural pothos, and the artificial pothos (*P* < 0.01). With regards to the ‘kind or unkind’ descriptor, the ‘peaceful or anxious’ descriptor, and the ‘pleasant or unpleasant’ descriptor, the fabric was significantly more ‘kind’, more ‘peaceful’, and more ‘pleasant’ than the metal, the natural pothos, and the artificial pothos (*P* < 0.01). Also, the fabric was significantly more ‘like’ than the metal and the natural pothos in the ‘like or dislike’ descriptor (*P* < 0.05).

**Table 2:** Summary of Steel-Dwass’s test results using the subjective SD method

**Adjectives**	**m-f**	**m-n**	**m-a**	**f-n**	**f-a**	**n-a**
warm, cold	m < f^**^		m < a^**^	f > n^**^	f > a^**^	–
smooth, rough	m > f^**^	m > n^**^	m > a^**^	f < n^*^	f < a^**^	–
soft, hard	m < f^**^	m < n^**^	m < a^**^		f > a^**^	–
natural, artificial	m < f^**^	m < n^**^	m < a^**^	–	–	
familiar, unfamiliar	–		–	–	–	–
kind, unkind	m < f^**^	–		f > n^**^	f > a^**^	–
calming, stimulating	–	–	–			
peaceful, anxious	m < f^**^			f > n^**^	f > a^**^	
pleasant, unpleasant	m < f^**^			f > n^**^	f > a^**^	
like, dislike	m < f^**^			f > n^**^	f > a^*^	

*, *P* < 0.05 by Steel-Dwass’s test; **, *P* < 0.01 by Steel-Dwass’s test; -, any difference of median without statistical significance; >, the left side is larger than the right side in the descriptor; < the right side is larger than the left side in the descriptor. m, metal; f, fabric; n, natural pothos; a, artificial pothos.

The metal was considered to be significantly colder than the other materials as opposed to the fabric, which was regarded as significantly warmer than the other materials. Similarly, the smooth surface of the metal was seen as significant compared with the rough surface of the fabric. With regard to the adjectival descriptors of soft and hard, the hardness of the metal was considered significant. On the contrary, the fabric was considered to be significantly softer than the artificial pothos, but not the natural pothos. There was a significant positive tendency shown in the adjectival descriptors for the fabric; ‘kind or unkind’, ‘peaceful or anxious’, ‘pleasant or unpleasant’, and ‘like or dislike’, however there was no significant difference in the others. No difference between artificial and natural pothos was seen in any of the adjectival descriptors.

The SD results indicate that the differences between the metal and the fabric were clearly recognized, but not the differences between the natural pothos and the artificial pothos. In addition, people tended to have a feeling of comfort from contact with a softer and warmer material.

### Cerebral hemodynamics

**Figure 5:**
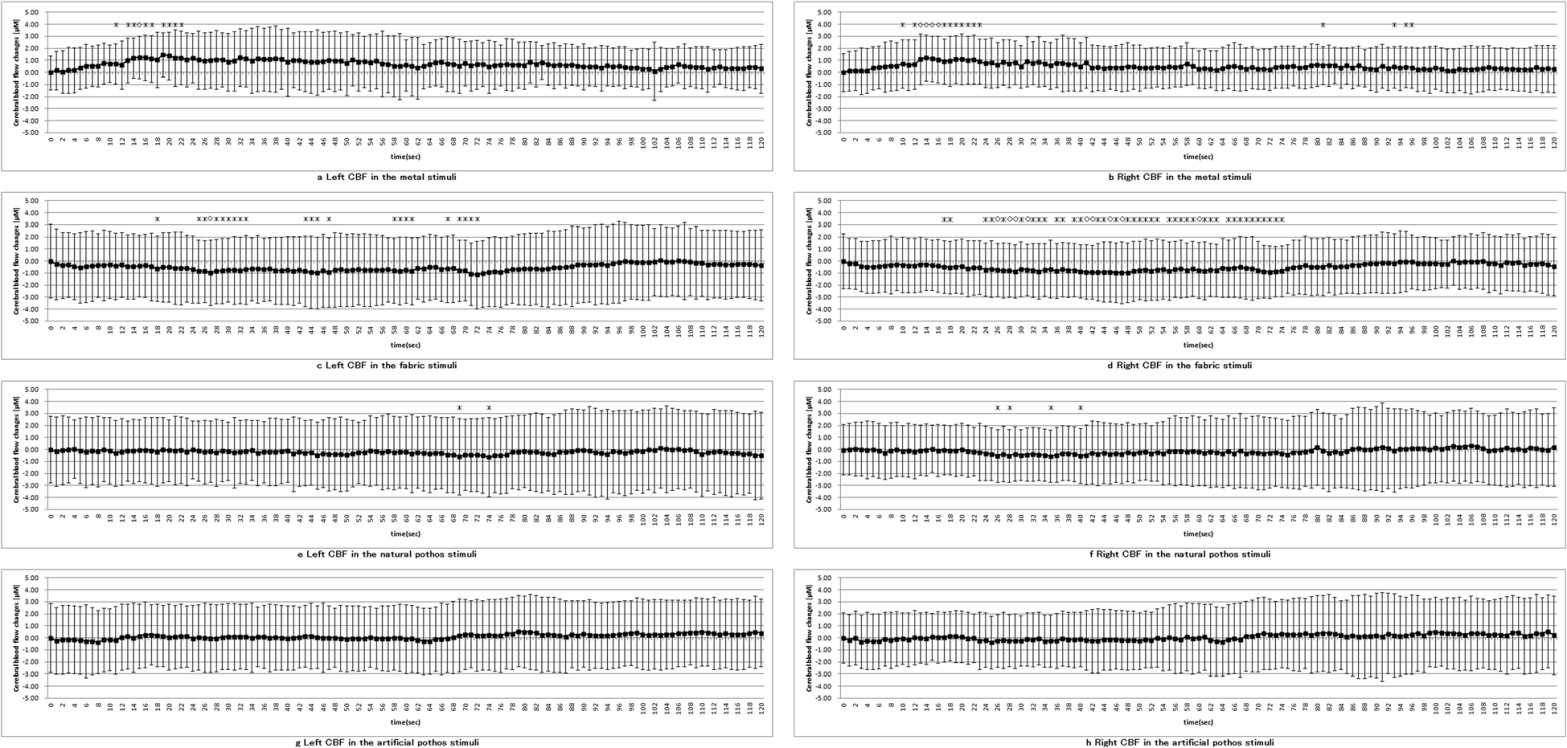
**Time-series variations in cerebral blood flow.** * *P* < 0.05 by Student’s *t* test compared with pre-stimulus (average for 30 s) ◇ *P* < 0.01 by Student’s *t* test compared with pre-stimulus (average for 30 s). CBF rose significantly immediately after touching the metal (**a, b**). Significant decreases in CBF were continuously seen between 10 s and 80 s with the fabric (**c, d**). For the natural pothos stimulus, there was a slight but significant reduction in CBF in the first half of the stimulus for the right frontal lobe and in the latter half of the stimulus (**e, f**) for the left frontal lobe. However, there was no significant difference in the stimulus of artificial pothos (**g, h**).

Figure 5 shows the time-series variations of CBF. As shown, the CBF of both frontal areas for the metal sample increase compared with the pre-stimulus results from 10 s to 20 s. Also, significant decreases of both prefrontal areas are continuously seen from 15 s to 75 s for the fabric sample. Moreover, the natural pothos sample showed significant decreases in the left prefrontal area around 70 s and in the right prefrontal area around 30 s. However, there was no significant difference in the stimulus of artificial pothos.

## Discussion

Some previous studies have shown that pleasant feelings from tactile stimuli mainly originate in the surface roughness of materials, whether the touch is active or passive. We know from our daily experiences that smooth and soft textures like velvet produce pleasant feelings, and rough and hard textures produce unpleasant feelings [46-49]. In this experiment, it was recognized that fabric stimulus created ‘soft’ and ‘kind’ impressions in the subjects as well as the feeling of being ‘comfortable’, as past studies have shown. Metal produced a neutral rating in the ‘pleasant or unpleasant’ descriptor, although it created a ‘hard’ impression, which induces relatively unpleasant emotions.

However, CBF in the orbitofrontal cortex (OFC) as a physiological index of ‘pleasantness or unpleasantness’ shows significant decrease in the fabric stimulus and significant increase in the metal stimulus.

There is currently some debate about the functional role of the prefrontal area with regards to tactile stimuli: Rolls [50] reported that there are different domains in the OFC, which work for pleasantness and unpleasantness, respectively; Hagen [51] detected the activity of neutral feeling valence in the OFC; and Kostopoulos [52] suggested that working memory was activated in the ventrolateral prefrontal cortex. Therefore, it is difficult to specify exactly what activity is taking place in the prefrontal area, because of the limitations of the NIRS, which has only two points of measurement. It has been proposed that the affective reaction caused by tactile stimuli is also found in domains other than the orbital prefrontal area [53]. However, it can be said that people showed a calming response physiologically to pleasant stimuli. This is shown in the physiologically significant reduction of oxygenated hemoglobin for the fabric stimulus in this experiment. On the contrary, a significant increase of oxygenated hemoglobin for the metal stimulus means that people feel physiological stress, although the results were neutral psychologically for the metal stimulus. The cause of this physiological stress is considered to be the cold stimulus when metal is touched. This was controlled in the laboratory, in order to eliminate the influence of differences in temperature of the materials. However, it is known that the warm-cold sense in touch is related to the conduction of heat between the skin and the materials touched [54]. The thermal conductivity of aluminum, used as the metal material in this experiment, is 236 W/mK [55], and is remarkably higher than that of fabric (0.16~0.32 W/mK) [56] or resin (0.08~0.42 W/mK) [57], and it may be considered that this caused the ‘cold’ impression, which is not evident in other materials.

Furthermore, the results from the natural pothos stimulus can also be discussed based on these findings. There was a slight, although statistically significant reduction in CBF, despite an unclear impression psychologically.

LeDoux [58] suggested a model of two pathways, which indicates that there are two amygdale pathways for arising affects; one is ‘low road’, from thalamus to amygdale directly, and the other is ‘high road’, from thalamus to amygdale via cortex. It is considered that the affect through the ‘high road’ would be conscious as subjective experiences, but the affect through the ‘low road’ would be expressed as physiological effects without recognition in this hypothesis. We could insist the existence of ‘low road’, when the directions of psychological and physiological reaction are different, or the stimulus cannot be recognized clearly. The natural pothos stimulus made a slight, although significant reduction in the amount of CBF, despite having left a weaker impression than the fabric and the metal psychologically and indicating neutral in the descriptor of ‘pleasant or unpleasant’ in our experiment. This could imply the existence of the affect arising through ‘low road’, or unconscious physiological reaction to the touch of a natural pothos. It is thought that the touch of a natural pothos cause an unconscious physiological calming reaction. This supports the results shown in the tactile experiments with wood of Miyazaki *et al.*[59] and Morikawa *et al.*[60].

Most studies of affective touch have been conducted by a psychophysical approach and the evaluation of impressions of ‘pleasantness or unpleasantness’ is made by verbal methodology while the subject is conscious. However, at present, it is generally agreed that cognition of the stimulus that causes emotions is not necessary for the emotions to occur [61]. That is to say, the findings in this experiment show that emotions also arise while unconscious. It seems that as a result of processing tactile stimuli of a leaf of natural pothos, people were made to produce physiological calmness from an unconscious state. The psychological index and the physiological index did not coincide in the previous study [60], and it has been shown that the physical reaction and the cognition of ‘pleasant or unpleasant’ emotions did not necessarily correspond.

According to the triune brain hypothesis proposed by MacLean [62], the brain has peculiar functions, which can be divided into three: a reptile’s brain (hypothalamus, and so on), the old mammal’s brain (limbic system), and a new mammal’s brain (cerebral neocortex). These peculiar functions depend on the evolutional stage and these three brains have a hierarchy [62]. Based on this concept of the hierarchy of the brain, if it is because of the information processing that determines how creatures adapt to their environment that pleasant or unpleasant emotions arise in a primitive reptile’s brain [63], then it is possible that the physiological reaction that arises from touching a leaf of the natural pothos may be an essential message from the primitive brain that plants are indispensable to the living environment of human beings.

## Conclusions

It was found that actively touching a leaf of natural pothos caused people to experience an unconscious calming response. This research supports the various previous studies that plants, nature, and material of natural origin bring feelings of relaxation to people. The results of this experiment might have been different if leaves with various different surface types had been used and it is necessary to examine this further. This report offers a new framework for understanding the relationship between human beings and plants or nature.

## Consent

Written informed consent was obtained from the subject for publication of this report and any accompanying images.

## Abbreviations

ANOVA: Analysis of variance; CAM: Complementary and alternative medicine; CBF: Cerebral blood flow; ERP: Event-related potentials; NIRS: Near infrared spectroscopy; OFC: Orbitofrontal cortex; SD: Semantic differential.

## Competing interests

The authors declare that they have no competing interests.

## Authors’ contributions

KK designed and carried out the study, performed the statistical analysis and drafted the manuscript. Administrative supervision was provided by IY. Both authors read and approved the final manuscript.
